# Targeting workload to ameliorate risk of heat stress in industrial sugarcane workers

**DOI:** 10.5271/sjweh.4057

**Published:** 2022-12-30

**Authors:** Rebekah AI Lucas, Bethany D Skinner, Esteban Arias-Monge, Kristina Jakobsson, Catharina Wesseling, Ilana Weiss, Scarlette Poveda, Fatima I Cerda-Granados, Jason Glaser, Erik Hansson, David H Wegman

**Affiliations:** 1 School of Sport, Exercise and Rehabilitation Sciences, University of Birmingham, Edgbaston, Birmingham, UK; 2 La Isla Network, Washington DC, USA; 3 Instituto Tecnológico de Costa Rica, Cartago, Costa Rica; 4 School of Public Health and Community Medicine, Sahlgrenska Academy, University of Gothenburg, Gothenburg, Sweden; 5 Department of Occupational and Environmental Medicine, Sahlgrenska University Hospital, Gothenburg, Sweden; 6 Unit of Occupational Medicine, Institute of Environmental Medicine, Karolinska Institutet, Stockholm, Sweden; 7 University of Massachusetts Lowell, Lowell MA, USA

**Keywords:** heart rate, heat exposure, industrial agriculture, intervention, observational study, physical workload

## Abstract

**Objective:**

The aim of this study was to quantify the physiological workload of manual laborers in industrial sugarcane and assess the effect of receiving a rest, shade, and hydration intervention to reduce heat stress exposure risk.

**Methods:**

In an observational study, physiological workload was evaluated for burned cane cutters (BCC), seed cutters (SC) and drip irrigation repair workers (DIRW) using heart rate (HR) recorded continuously (Polar®) across a work shift. Workers’ percentage of maximal HR (%HR_max_), time spent in different HR zones, and estimated core temperature (ECTemp) were calculated. The effect of increasing rest across two harvests was evaluated for BCC and SC.

**Results:**

A total of 162 workers participated in this study [52 BCC (all male), 71 SC (13 female) and 39 DIRW (16 female)]. Average %HR_max_ across a work shift was similar between BCC and SC (BCC: 58%, SC: 59%), but lower in DIRW (51%). BCC and SC spent similar proportions of work shifts at hard/very hard intensities (BCC: 13%, SC: 15%), versus DIRW who worked mostly at light (46%) or light-moderate (39%) intensities. SC maximum ECTemp reached 38.2°C, BCC 38.1°C; while DIRW only reached 37.7°C. Females performed at a higher %HR_max_ than males across work shifts (SC 64% versus 58%; DIRW 55% versus 49%). An additional rest period was associated with a lower average %HR_max_ across a work shift in BCC.

**Conclusion:**

In this setting, BCC and SC both undertake very physiologically demanding work. Females maintained a higher workload than male co-workers. Regulated rest periods each hour, with water and shade access, appears to reduce physiological workload/strain.

Occupational heat stress has multiple well-established adverse health outcomes including heat illness, heat stroke, increased accident risk, and reduced work capacity and productivity (1). Recently occupational heat stress has been linked to kidney injury and chronic kidney disease of non-traditional origin (CKDnT; unrelated to diabetes and hypertension) due to the occupational nature of CKDnT and its association with strenuous manual labor in hot environments (2–4). In Central America, tens of thousands of young male agricultural workers, particularly sugarcane cutters, have died of CKDnT since the disease emerged in the 1970s (4–6). Concurrently, sugarcane production in Central America has significantly increased, yet the sugarcane industry still operates in a low regulatory environment with little organized labor (7). Effective occupational heat stress prevention in this region and within the sugarcane industry is imperative and overdue (4), especially considering the CKDnT epidemic and climate change-related increases in global temperatures (8).

In 2017 we began a multi-year cohort study assessing an intervention program for workers at the Ingenio San Antonio (ISA) sugar mill in Chichigalpa, Nicaragua (*The Adelante Initiative; PREP*) (9). This intervention program was designed to reduce the risk of excessive heat exposure for manual labourers engaged in sugarcane agriculture by implementing regulated rest periods, providing shade for rest close to worksites (eg, moveable tents) plus cool and portable water and electrolyte solutions that workers had easy access to in the field throughout their workday. Our initial investigations showed that workers considered to have the highest workload were at the greatest risk of kidney injury (9). We also observed that work practices preventing heat stress should be strengthened for burned sugarcane cutting *and* for other physically demanding jobs (eg, seed cutting). Therefore, we endeavored to quantify physiological workload in a range of manual jobs performed by workers at ISA to identify jobs where workers were at increased risk of excessive heat exposure and in need of more rigorous workplace intervention.

Workload can be measured in occupational field settings using a variety of methods including, subjective methods (eg, visual analog scales or interviews), observation, motion capture (eg, accelerometers), heart rate and oxygen consumption (ISO 8996) (10). Heart rate (HR) monitoring provides a pragmatic and accurate means of assessing physiological workload with high temporal resolution in field settings because of the strong positive linear relationship HR has with oxygen consumption (11, 12). Furthermore, sequential HR measures can be used to estimate core body temperature, a critical heat strain component (13).

## Aims

The primary aim of this study (Aim 1) was to assess and compare physiological workload using continuous HR monitoring in addition to observations for three different outdoor jobs – burned cane cutters (BCC), seed cutters (SC) and drip irrigation repair workers (DIRW) – performed at an industrial sugarcane mill. These jobs were selected because workers and management reported burned cane cutting to be the most strenuous and difficult job at the mill; senior occupational physicians performing work observations found seed cutting to be a very physically demanding job, with initial Adelante studies showing SC were at high risk of excessive heat exposure and kidney injury (9); in comparison, DIRW performed a less physically demanding job and were at low risk for kidney injury (9, 14).

The subsidiary aim (Aim 2) was to assess if an enhanced rest, shade, hydration (RSH) intervention, designed to address heat stress exposure in industrial agriculture, reduces workers’ physiological workload. To address this aim, data collected across two harvests were compared among BCC and SC as these workers were identified as being at high risk of excessive heat exposure and kidney injury and therefore, most likely to benefit from the RSH intervention.

## Methods

### Study population

*The Adelante Initiative* at the ISA sugarcane mill in Chinandega, Nicaragua, started in 2017 (our first observed harvest, H1) and is ongoing. Data collection for the present study occurred during the 2018–19 and 2019–20 sugarcane harvests (denoted H2 and H3, respectively). Workload data for eight common job categories were collected and are reported in the supplementary material www.sjweh.fi/article/4057 (appendices 1 and 2). To address Aim 1, an observational study was conducted during H3 comparing three jobs (BCC, SC and DIRW). To address Aim 2, data collected in BCC and SC during H2 were compared to data collected in H3.

DIRW, SC and BCC job tasks were routine and repetitive day-to-day, with little variation throughout the harvest period. BCC and SC were paid piecework wages, while DIRW were paid a day rate. BCC use mill-provided machetes to cut the cane (burned the night before) at ground level, topping the cane stalks, and piling the cane by hand. SC use mill-provided machetes to cut green cane, removing the cane greenery and cutting the unburned cane into smaller lengths (as defined by the mill). These stripped lengths are then gathered into bundles and packed in sacks or tied together with cane greenery. DIRW are responsible for finding and repairing leaks in the mill’s drip irrigation system, requiring them to walk through fields and, if a leak is found, dig out a section of PVC pipe and repair it. Participants in the current study were recruited from workers already participating in a larger cross-harvest study, with an effort made to match the larger workforce’s demographics. See Glaser et al (14) and Hansson et al (9) for a detailed description of the study context and data collection of this larger study.

Physiological workload assessments were conducted mid harvest (Jan–March) or at the end of harvest (April). To provide a robust estimate of physiological workload for each job category, 2–3 workdays were observed with 5–20 workers per workday. Immediately before they started their workday, workers were equipped with a Polar® HR monitor strapped to the chest. When workers finished their workday, they completed a brief questionnaire, and the HR monitor was removed. During work shifts, occupational hygienists and senior occupational physicians recorded qualitative work observations.

### The intervention

Ahead of the 2018–2019 harvest (H2) all jobs were recommended to have a first 10-minute rest period at 08:00 hours (as work starts at sunrise, ~06:00 hours), reflecting the need for a morning break while meeting the workers’ desire to take advantage of the cooler morning hours. For subsequent hourly rest periods, BCC were recommended to have longer rests (15–20 minutes) over the course of the day, while other jobs were recommended to have 10-minute hourly rests. Ahead of the 2019–2020 harvest (H3), all jobs were recommended to have an earlier first rest period at 07:00 hours (10-minute rest for BCC and SC, 5 minutes for DIRW), following H2 assessments that showed workers performed at high work intensities during the early morning (see H2 physiological workload data; figure 3). Additional intervention recommendations are detailed in Glaser et al (7, 14). Briefly: shade tents and fluid reservoirs were moved throughout the day to be ≤50 meters from every worker throughout the workday, shade tents were rotated so that closed sides continued to face the sun, and efforts were made to improve the taste of supplementary electrolyte rehydration fluids.

### Measures and variables

Wet-bulb globe temperature was recorded in the field alongside workers across their workday via portable weather stations (QuesTemp® 34, 3M; Kestrel 5400 Heat Stress Tracker, Nielsen-Kellerman). Heart rate was continuously recorded (beat-to-beat) from a sensor fitted on a chest strap (RC3X or Polar Team Pro Sensor, Polar Electro, Kempele, Finland). HR data are expressed as percentage of maximal HR (%HR_max_), with a regression equation used to predict HR_max_ (208 - 0.7 × age) (15). HR data were used to calculate the observed rest:work ratio for each worker, with a rest period defined as a drop in HR >10 bpm lasting ≥4 minutes. Physiological workload was categorized based on %HR_max_ as: maximal (91–100%); very hard (81–90%); hard (71–80%); moderate (61–70%); light moderate (51–60%); and light (≤50%).

Sequential HR measurements were also used to calculate estimated core temperature (ECTemp) (13) as an indicator of heat strain (ie, the effect of heat stress on the body) (16). For each worker, time spent at an ECTemp ≥38 °C was calculated to reflect international occupational health and safety standards that recommend workers avoid core body temperature ≥38 °C (17, 18).

### Data analysis

**Figure 1:**
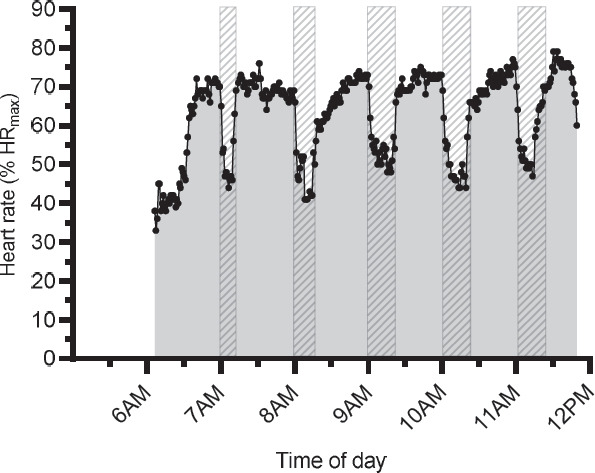
An example tracing of physiological workload (%HR_max_) across the work shift for one worker. Striped boxes indicate the observed rest periods, alongside the corresponding decrease in heart rate. The grey shaded area indicates the area quantified for area under the curve (AUC) analysis. Daily calculations of AUC include both work and rest periods.

Outcome measures are presented as mean values and 95% confidence intervals (CI) for all work groups unless otherwise stated. HR was sampled at 0.1-second intervals, then exported and aggregated into 1-minute averages using MATLAB (MathWorks Inc, Natick, MA, USA). HR outliers (eg, abnormal recordings >220 beats/minute) were removed. The rest:work ratio was calculated comparing across a work shift the average amount of time spent resting (minutes) to the average time spent working (minutes). For example, a rest:work ratio of 14:40 represents 14 minutes of rest for every 40 minutes of work performed across the full shift. To assess cumulative workload, area under the curve (AUC) was calculated for HR across the full work shift (AUC %HR_max_ as shown in figure 1) as well as across the first five hours of the work shift.

## Results

### Aim 1: Physiological workload comparisons between jobs

*Context*. Data collected during H3 was used to address Aim 1. In H3, workload was assessed in 40 male BCC [33 (95% CI 31‒36) years] across two days; in 58 SC [29 (95% CI 28‒31) years, 10 females] across three days; and in 39 DIRW workers [29 (95% CI 26‒31) years, 16 females] across two days.

During H3, daily average wet-bulb globe temperature in the field were similar between jobs on data collection days [BCC: 26.7 (95% CI 25.6‒27.9) °C; SC: 28.6 (95% CI 26.9‒30.2) °C; DIRW: 27.5 (95% CI 25.7‒29.4) °C].

On data collection days, work shift duration for BCC was approximately one hour shorter than for SC and approximately two hours shorter than DIRW (table 1a). During a work shift, BCC and SC had a similar number and duration of rest periods, with both BCC and SC having longer rest periods compared to DIRW (table 1a). The rest:work ratio (minutes) for BCC was 14:40 (26% rest), compared to 14:51 (22% rest) for SC and 12:59 (17% rest) for DIRW.

**Table 1a:** Work shift context, and physiological workload (based on heart rate) for burned cane cutters (BCC), seed cutters (SC) and drip irrigation repair workers (DIRW) across the work shift during Harvest 3. Data presented as mean (95% confidence intervals). [%HR_max_=percentage of maximal HR; CI=confidence interval.]

	Work shift context					Physiological workload	
	
	Work shift duration	Rest periods		Work periods		Average %HR_max_ work + rest periods	Average %HR_max_ work periods
				
	Mean (95% CI)	Mean (95% CI)		Mean (95% CI)		Mean (95% CI)	Mean (95% CI)
				
	Hours	N	Minutes	N	Minutes	%	%
BCC (N=40)	05:38 (05:31–05:45)	5 (5–5)	14 (13–15)	6 (6–6)	40 (39–42)	58 (56–60)	64 (62–65)
SC (N=58)	06:39 (06:25–06:52)	5 (5–5)	14 (13–16)	6 (6–6)	51 (48–53)	59 (58–60)	63 (62–64)
DIRW (N=39)	07:41 (07:39–07:43)	5 (5–6)	12 (11–13)	6 (6–7)	59 (53–65)	51 (50–53)	53 (51–54)

### Physiological workload

**Figure 2:**
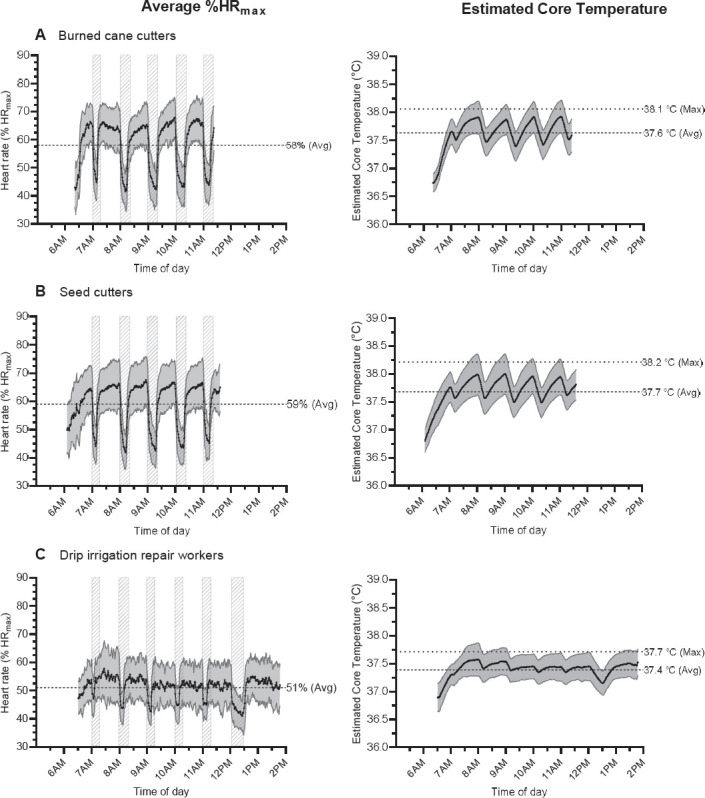
Average heart rate (% HR_max_) (left panel) and estimated core temperature (right panel) across the work shift for (A) burned cane cutters (N=40), (B) seed cutters (N=58), and (C) drip irrigation repair workers (N=39) during the 2019–2020 harvest. Grey striped boxes indicate the recommended rest periods. Dashed (- - -) and dotted (· · ·) lines indicate averages and maximums, respectively. Note: estimated core temperature was calculated from sequential HR measurements.

Average %HR_max_ across a work shift was similar between BCC and SC, both working at a higher intensity when compared to DIRW (figure 2, table 1a). BCC and SC spent >10% of their shift working at a hard intensity, while DIRW spent very little of their shift working at a hard intensity (table 1b).

**Table 1b:** Proportion of the work shift spent at relative work intensities (inclusive of work and rest periods) for burned cane cutters (BCC), seed cutters (SC) and drip irrigation repair workers (DIRW) across the work shift during Harvest 3. [%HR_max_=percentage of maximal HR; CI=confidence interval.]

	Proportion of work shift spent at relative work intensities											

	Light <50 %HR_max_		Light – moderate 51–60 %HR_max_		Moderate 61–70%HR_max_		Hard 71–80 %HR_max_)		Very hard 81–90 %HR_max_		Maximal 91–100 %HR_max_	
					
	Mean (95% CI)		Mean (95% CI)		Mean (95% CI)		Mean (95% CI)		Mean (95% CI)		Mean (95% CI)	
					
	%	Minutes	%	Minutes	%	Minutes	%	Minutes	%	Minutes	%	Minutes
BCC (N=40)	26 (22–29)	87 (74–99)	24 (20–28)	80 (67–94)	37 (32–42)	125 (107–143)	12 (8–17)	42 (27–56)	1 (0–3)	4 (0–9)	0 (0–0)	0 (0–0)
SC (N=58)	23 (20–26)	87 (76–98)	31 (27–35)	117 (102–132)	32 (28–35)	127 (111–144)	14 (10–17)	61 (45–78)	1 (0–2)	6 (2–10)	0 (0–0)	0 (0–1)
DIRW (N=39)	46 (38–55)	215 (173–257)	39 (33–45)	180 (153–207)	13 (8–18)	58 (35–81)	2 (0–3)	8 (3–13)	0 (0–0)	0 (0–0)	0 (0–0)	0 (0–0)


Across the first five hours of the work shift, AUC %HR_max_ was similar between BCC [17 509 (95% CI 16 861‒18 157)] and SC [17 915 (95% CI 17 468‒18 362)], with both showing a greater AUC %HR_max_ when compared to DIRW [15 509 (95% CI 15 004‒16 015)]. However, AUC %HR_max_ across the full work shift was lower for BCC [19 634 (95% CI 18 911‒20 357)] when compared to both SC [23 480 (95% CI 22 340‒24 619)] and DIRW (23 569 (95% CI 22 832‒24 305)], reflecting differences in job-specific work shift durations. Across the full work shift, AUC %HR_max_ was similar between SC and DIRW despite SC having a shorter work shift than DIRW.

SC and DIRW included both male and female workers, while BCC were all males. Female SC worked at a higher %HR_max_ than male SC across a work shift [inclusive of work and rest periods; female: 64 (95% CI 60‒67) %HR_max_ versus male: 58 (57‒59) %HR_max_] and during work periods only [female: 67 (95% CI 64‒70) %HR_max_ versus male: 62 (95% CI 61‒64) %HR_max_]. Similarly, female DIRW worked at a higher %HR_max_ than male DIRW across a work shift [inclusive of work and rest periods; female: 55 (95% CI 53‒58) %HR_max_ versus male: 49 (95% CI 47‒51) %HR_max_] and during work periods only [female: 56 (95% CI 54‒58) %HR_max_ versus male: 50 (49‒52) %HR_max_].

### Estimated core temperature

SC reached a similar maximum ECTemp [38.2 (95% CI 38.1‒38.3) °C] as BCC [38.1 (95% CI 38.0‒38.2) °C] with both jobs reaching a greater ECTemp when compared to DIRW [37.7 (95% CI 37.6‒37.8) °C). Average ECTemp was similar in BCC [37.6 (95% CI 37.5‒37.7) °C] and SC [37.7 (95% CI 37.6‒37.7) °C], but greater when compared to DIRW [37.4 (95% CI 37.3‒37.5) °C] (see also figure 2).

SC [21 (95% CI 14‒27) %, range: 0–89%] spent a similar proportion of their work shift at an ECTemp >38 °C when compared to BCC [15 (95% CI 9‒21) %, range: 0–56%], with both spending a greater proportion compared to DIRW [1 (95% CI 0‒2) %, range: 0–11%].

Female SC reached a higher maximum ECTemp than their male counterparts [female: 38.4 (95% CI 38.3–38.6) °C versus male: 38.2 (95% CI 38.1–38.2) °C], while maximum ECTemp was similar between female and male DIRW [female: 37.8 (95% CI 37.7–37.9) °C versus male: 37.6 (95% CI 37.5–37.8) °C]. Female SC [42 (95% CI 21–63) %] spent a greater proportion of their work shift at an ECTemp >38 °C compared to male SC [16 (95% CI 10–22) %]. Male and female DIRW spent a similar proportion of the work shift at an ECTemp >38 °C [1 (95% CI 0–3) % and 1 (95% CI 0–2) %, respectively).

### Aim 2: Rest, share, hydration intervention evaluation

*Context*. To address aim 2, data collected during H2 in 12 male BCC [37 (95% CI 31–42) years, across two days] were compared to H3 BCC data (participant characteristics above) and data collected during H2 in 13 SC [28 (95% CI 27–29) years, 3 females, across two days] were compared to H3 SC data.

For BCC, daily average wet-bulb globe temperatures on data collection days were higher during H2 compared to H3 [H2: 29.5 (95% CI 28.4–30.5) °C versus H3: 26.7 (95% CI 25.6–27.9) °C]. For SC, daily average wet-bulb globe temperatures were more similar on data collection days [H2: 30.1 (95% CI 28.9–31.3) °C; H3: 28.6 (95% CI 26.9–30.2) °C].

For BCC, total rest was designed to increase from 65 minutes in H2 to 80 minutes in H3 for their 6-hour work shift. On data collection days, the observed rest:work ratio (minutes) for BCC increased slightly from 13:50 (21% rest) in H2 to 14:40 (26% rest) in H3 (table 2). For SC, total rest was designed to increase from 70 minutes in H2 to 100 minutes in H3 for their 8–9-hour work shift. However, only work shift data until the 12:00 hour rest period was used as all SC work shifts ended at ~midday in H2. For SC, there was an increase in the observed rest:work ratio from 9:67 (13% rest) in H2, to 12:47 (20% rest) in H3 (up until ~midday).

**Table 2:** Work shift context, physiological workload (based on heart rate) for burnt cane cutters (BCC) and seed cutters (SC) before (Harvest 2; H2) and after (Harvest 3; H3) implementation of an enhanced intervention. [%HR_max_=percentage of maximal heart rate; CI= confidence interval.]

		Work shift duration	Rest periods		Work periods		Average %HR_max_ work + rest periods	Average %HR_max_ work periods
				
		Mean (95% CI)	Mean (95% CI)		Mean (95% CI)		Mean (95% CI)	Mean (95% CI)
				
		Hours	N	Minutes	N	Minutes	%	%
BCC	H2 (N=12)	05:46 (05:26–06:06)	4 (3–4)	13 (12–15)	5 (4–5)	50 (47–53)	63 (60–65)	68 (65–71)
	H3 (N=40)	05:38 (05:31–05:45)	5 (5–5)	14 (13–15)	6 (6–6)	40 (39–42)	58 (56–60)	64 (62–65)
SC^a^	H2 (N=13)	05:42 (05:20–06:04)	3 (3–4)	9 (7–10)	4 (4–5)	67 (58–75)	60 (57–64)	63 (60–66)
	H3 (N=58)	05:42^a^ (05:36–05:48)	5 (5–5)	12 (12–13)	6 (6–6)	47 (46–48)	60 (59–62)	63 (62–65)

^a^ To account for shorter work shift (ending ~midday) for SC in H2, H3 data were only examined up to the 12:00 hour rest period for between harvest comparisons.

### Physiological workload

**Figure 3:**
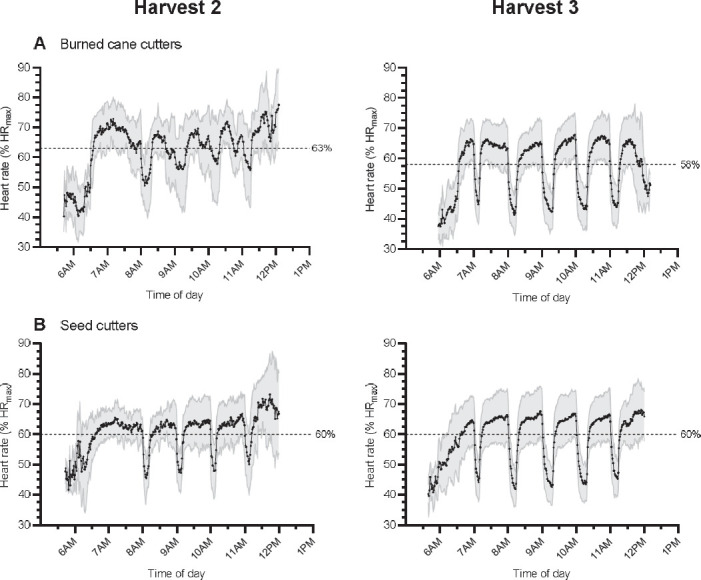
Average heart rate (%HRmax) across the work shift for A) Burned Cane Cutters and B) Seed Cutters before (Harvest 2) and after (Harvest 3) implementation of an enhanced intervention. Dashed lines indicate the average % HRmax.

For BCC, average %HR_max_ across the work shift was lower in H3 when compared to H2 (figure 3, table 2). For SC, average %HR_max_ across the work shift was similar between H2 and H3 (figure 3, table 2).

### Estimated core temperature

For BCC, maximum ECTemp [H2: 38.3 (95% CI 38.1–38.4) °C; H3: 38.1 (95% CI 38.0–38.2) °C] and average ECTemp [H2: 37.8 (95% CI 37.6–37.9) °C; H3: 37.6 (95% CI 37.5–37.7)°C] did not significantly differ between H2 and H3. The proportion of the work shift spent at an ECTemp >38 °C did not significantly differ for BCC during H2 [30 (95% CI 15–45) % and H3 [15 (95% CI 9–21) %]. For SC, both maximum ECTemp [H2: 38.3 °C [95% CI 38.0–38.5]; H3: 38.2 °C [95% CI 38.1–38.3]) and average ECTemp [H2: 37.8 (95% CI 37.6–37.9) °C; H3: 37.7 (95% CI 37.7–37.8) °C] remained similar between H2 and H3. The proportion of the work shift spent at an ECTemp >38 °C was similar for SC during H2 [23 (95% CI 7–39) %] and H3 [22 (95% CI 16–29) %].

### Discussion

This study aimed to quantify physiological workload in three different outdoor manual jobs within the sugarcane industry and assess how an enhanced RSH intervention affects workers’ physiological workload. One key finding was that SC perform as physically demanding work as BCC, indicating that SC should be afforded similar worker protections. Secondly, this study provides evidence that in BCC physiological workload intensity was reduced when break frequency and duration were enhanced.

### Aim 1: Physiological workload comparisons between jobs

In the current study, both BCC and SC worked at similar relative intensities (~58%HR_max_) across their work shift. These quantitative data support our earlier qualitative observations that seed cutting is a very physically demanding job within industrial sugarcane agriculture (9, 14). In these previous studies, BCC and SC also demonstrated an elevated risk of kidney injury over the harvest (9, 14). Thus collectively, our studies indicate that workers with the highest workload, ie, working repeatedly at hard or very hard intensities during their work shift and subject to higher ECTemp, are the most at risk of developing kidney injury over a harvest. Somewhat to our surprise, SC in the current study reached similar ECTemp as BCC, with both jobs spending a similar amount of time at or >38 °C, the level that international health and safety standards recommend workers avoid (17, 18). These data indicate that work demands for BCC are not unique and that other manual jobs in industrial sugarcane production require physiological workload assessment and appropriate workplace protections to reduce/limit workers’ exposure to excessive heat stress.

On H3 data collection days, RSH recommendations were followed and appeared to create strong rest:work norms in the observed BCC and SC (see 95% CI in table 1a). This is in contrast with uneven adherence in H2 where field monitoring indicated intervention implementation varied between work squads (7). Effective intervention implementation in industrial agricultural may demand continuous monitoring and prioritization to ensure workers protections are upheld. Despite the intervention, BCC in the current study spent 74% of their shift (4:11 hours out of their 5:38 hour work shift) working at ≥50% of their HR_max_. In a previous study from El Salvador, we showed that BCC spent over half their shift and a similar absolute amount of time (4:44 hours out of their 7:30 hour work shift) working at ≥50% of their HR_max_ (19). Notably, no formal RSH intervention was in place at the time of workload data collection in El Salvador and workers were responsible for their own water supply and rest/shade schedule (20). Collectively, these data indicate that shorter work shifts plus more frequent and regulated rest periods do not, alone, circumvent the strenuous and physically demanding nature of the work. Indeed, BCC and SC spent up to 46 and 67 minutes working at hard and very hard exercise intensities (≥71%HR_max_). This propensity to work at higher physical intensities may be driven by the piecework payment system used throughout industrial agriculture (21, 22). Indeed, our own field observations show some workers continue to work during rest periods to accomplish piece-rate goals (23).

Females formed part of the SC cohort and were shown to work at a higher physiological workload than their male counterparts. This higher relative work intensity in females is likely the result of recognized sex-differences in aerobic capacity. Consequently, females had to work harder to meet production targets. Long-term health and economic implications for female workers in our cohort and in industrial agriculture are unclear. However, it is likely that female workers will be disproportionately affected by the negative consequences of a piecework payment system (24, 25). Furthermore, these data indicate that females do perform physically demanding jobs within the sugarcane industry, are not precluded from occupational heat stress and hence, its association with CKDnT.

### Aim 2: Rest, shade, hydration intervention evaluation

The current study aimed to assess if increasing the rest component in the RSH intervention (adding an earlier rest period at 07:00 hours and extending rest periods to 15–20 minutes), reduced physiological workload. In BCC, increasing the rest component lowered the relative work intensity across the entire work shift *and* during work periods. Furthermore, this additional rest tended to reduce heat strain (indicated by a reduction in ECTemp). These promising findings appear to indicate that physiological workload and strain were reduced in BCC across their work shift when break frequency and duration were enhanced (see figure 3). However, the observational nature of these data, the low *n* in Harvest 2, plus the marginally lower daily average wet-bulb globe temperature values on data collection days in H3 versus H2, tempers conclusions drawn from these data alone. An experimental laboratory study could more fully test the impact associated with enhancing the rest components in a RSH intervention.

In contrast to BCC, physiological workload in SC did not change between H2 and H3 (up until ~midday), despite recommendations to increase total rest during H3. For SC, all work shifts in H2 ended at ~midday. This was due to management calling SC back to the mill at midday for Human Resource purposes. Typically, SC shifts in H2 and H3 ended between 13–14:00 hours, as our research team observed and SC and management reported at ISA. The shorter shifts in H2 likely affected SC workload comparisons between H2 and H3 and could explain why increasing the rest component in H3 did not reduce the physiological workload in this group (ie, there was a lower production target for shorter work shifts). It is also worth noting that for SC a formal RSH intervention was only recently implemented (since 2018), compared to BCC who for many years have had some type of workplace intervention in place (7). Thus, intervention adherence in the SC may be affected by perceptions on how the intervention affects production, which can take time to assuage (23).

### Methodological considerations

The current study used continuous heart rate measures to quantify physiological workload. Heart rate is a marker of physiological strain that can be used to quantify an individual’s metabolic workload and level of heat strain (10). Heat stress affects the linearity of the HR-metabolic work relationship, determining that under heat stress conditions HR may overestimate metabolic work (26). Thus, in the current study we have not used heart rate as a marker of metabolic work but rather physiological workload/strain.

Predicted body core temperature (ECTemp) was calculated using a validated model, which has been shown to have a slight overall bias of –0.03±0.32 °C (27). This determines that ECTemp values reported in the current paper may slightly overestimate workers’ actual body core temperature. However, this does not change ECTemp comparisons between jobs or between H2 and H3.

Future studies examining the cumulative strain associated with workload, could use AUC calculations, as utilized in this paper.

The work shift duration for SC was atypically short on three (two in H2 and one in H3) data collection days, with workers finishing by midday instead of between 13–14:00 hours. This highlights some of the challenges associated with data collection in the field where scientific requirements are subordinate to company requirements/demands.

### Concluding remarks

Quantitative occupational exposure assessments, such as physiological workload via continuous heart rate monitoring, are essential to identifying exposure risk for workers as well as the efficacy of workplace interventions. The current study indicates that burned cane cutting and seed cutting in industrial sugarcane production are very physically demanding jobs that require workers to periodically work at hard or very hard intensities and subjects them to heat strain. Notably, females also undertake physically demanding jobs within this industry and, in this study, experienced a higher workload intensity than their male co-workers. The current study also provides evidence that in BCC physiological workload intensity (as measured by HR%_max_) was reduced when the frequency and duration of rest periods (with easy access to shade and cool water) were enhanced. However, shorter work shifts plus more frequent and regulated rest periods cannot alone circumvent the strenuous and physically demanding nature of this piece-rate work.

### Funding

The *Natural Environment Research Council (*NE/T013702/1), La Isla Network, and the University of Birmingham funded this study.

PREP “Protection Resilience Efficiency and Prevention for workers in industrial agriculture in a changing climate” was funded by Forte (Sweden); NOAA (USA), NSF (USA) and UKRI (UK).

The Adelante study was funded by the Stavros Niarchos Foundation (GL 2016) and the German Investment Corporation (DEG/BSS Technical Assistance contract: F0877/SAP3705) and German Ministry for Economic Development and Cooperation (DEG/BMZ develoPPP-Performance Contract: 20190807), and the Ingenio San Antonio (ISA) sugar mill via the DEG and BMZ’s DeveloPPP.de Program. The intervention parts of these studies (eg, shade tents, water containers) were provided by the sugarcane mill where the study was carried out. Neither the company nor other sponsors had any role in the design, execution, interpretation, or writing of the study.

### Conflict of interest

The authors declare that the research was conducted in the absence of any commercial or financial relationships that could be construed as a potential conflict of interest.

### Ethical approval

This study was approved by the Comité de Ética para Investigaciones Biomédicas (CEIB), Facultad de Ciencias Médicas, Universidad Nacional Autónoma de Nicaragua (UNAN- León), FWA000045231/IRB00003342. Our trained staff apprised all workers of the study objectives and procedures and answered any questions before participants signed an informed consent.

## Supplementary material

Supplementary material

## Data Availability

The authors confirm that the data supporting the findings of this study are available within the article and its supplementary materials.
