# Supplementary material for: Targeting workload to ameliorate risk of heat stress in industrial sugarcane workers
Source: Scand J Work Environ Health. 2022 Dec 30;49(1):43–52. doi: 10.5271/sjweh.4057 (PMC10549916; doi:10.5271/sjweh.4057)
Supplement: Supplementary material [file SJWEH-49-43-S001.pdf]

Targeting workload to ameliorate risk of heat stress in industrial sugarcane workers[1]

by Rebekah AI Lucas,[2] Bethany D Skinner, Esteban Arias-Monge, Kristina Jakobsson, Catharina Wesseling, Ilana Weiss, Scarlette Poveda, Fatima I Cerda-Granados, Jason Glaser, Erik Hansson, David H Wegman

1. Supplementary material
2. Correspondence to: Rebekah AI Lucas, School of Sport, Exercise and Rehabilitation Sciences, University of Birmingham, Edgbaston, Birmingham, B15 2TT, United Kingdom. [E-mail: r.a.i.lucas@bham.ac.uk]

Appendix 1: Work shift context and physiological workload based on heart rate (HR) for the eight observed jobs during the 2020 harvest (H3). Burned cane cutters (n=40, across two work days), seed cutters (n=60, across three workdays), drip irrigation repair workers (n=39, across two work days), semi-mechanised seeders in dry soil (n=15, one work day), semi-mechanised seeders in moist soil (n=19, one work day), gravity irrigators (n=9, across two work days), semi-mechanised reseeders (n=40, across two work days), and weeders (n=20, one work day). Workers performing semi-mechanised seeding in moist soil had shorter evening shifts, often with no rest periods. Gravity irrigation workers had no discernible rest periods for the two observed workdays in H3. Data presented as mean [95% confidence intervals].

	**Work shift duration**	**Rest periods**		**Work periods**		**Average %HR$_{max}$, work periods**
	Mean (95% CI)	Mean (95% CI)		Mean (95% CI)		Mean (95% CI)
	hours	#	mins	#	mins	%
**Burned cane cutters**	05:38 (05:31–05:45)	5 (5–5)	14 (13–15)	6 (6–6)	40 (39–42)	64 (62–65)
**Seed cutters**	06:39 (06:25–06:52)	5 (5–5)	14 (13–16)	6 (6–6)	51 (48–53)	63 (62–64)
**Drip irrigation repair workers**	07:41 (07:39–07:43)	5 (5–6)	12 (11–13)	6 (6–7)	59 (53–65)	53 (51–54)
**Semi-mechanised seeders in dry soil**	06:39 (06:04–07:13)	6 (5–6)	10 (9–11)	7 (6–7)	49 (46–52)	68 (66–71)
**Semi-mechanised seeders in moist soil**	03:40 (03:32–03:47)	-	-	-	-	-
**Gravity Irrigators**	09:44 (08:36–10:52)	-	-	-	-	-
**Semi-Mechanised reseeders**	07:48 (07:38–07:58)	5 (4–5)	10 (10–11)	6 (5–6)	71 (66–76)	54 (52–56)
**Weeders**	07:29 (07:21–07:37)	5 (4–5)	18 (14–22)	6 (5–6)	59 (53–64)	57 (55–59)

Appendix 2: The percentage of the work shift and the average time spent at different work intensities (%HRmax) across a workday (inclusive of work and rest periods) for the eight observed jobs during the 2020 harvest (H3). Data presented as mean [95% confidence intervals].

	**Light** (<50 %HR_{max})		**Light – Moderate** (51-60 %HR_{max})		**Moderate** (61-70 %HR_{max})		**Hard** (71-80 %HR_{max})		**Very Hard** (81-90 %HR_{max})		**Maximal** (91-100 %HR_{max})	
	Mean (95% CI)		Mean (95% CI)		Mean (95% CI)		Mean (95% CI)		Mean (95% CI)		Mean (95% CI)	
	%	mins	%	mins	%	mins	%	mins	%	mins	%	mins
**Burned cane cutters**	26 (22–29)	87 (74–99)	24 (20–28)	80 (67–94)	37 (32–42)	125 (107–143)	12 (8–17)	42 (27–56)	1 (0–3)	4 (0–9)	0 (0–0)	0 (0–0)
**Seed cutters**	23 (20–26)	87 (76–98)	31 (27–35)	117 (102–132)	32 (28–35)	127 (111–144)	14 (10–17)	61 (45–78)	1 (0–2)	6 (2–10)	0 (0–0)	0 (0–1)
**Drip irrigation repair workers**	46 (38–55)	215 (173–257)	39 (33–45)	180 (153–207)	13 (8–18)	58 (35–81)	2 (0–3)	8 (3–13)	0 (0–0)	0 (0–0)	0 (0–0)	0 (0–0)
**Semi-mechanised seeders in dry soil**	7 (4–10)	27 (15–40)	16 (13–20)	63 (51–75)	41 (32–49)	161 (123–199)	31 (20–42)	127 (77–177)	5 (1–9)	20 (5–36)	0 (0–0)	0 (0–0)
**Semi-mechanised seeders in moist soil**	18 (12–23)	41 (29–52)	16 (11–21)	35 (24–45)	28 (22–34)	61 (46–76)	31 (22–41)	68 (47–88)	7 (2–12)	15 (5–25)	0 (0–1)	1 (0–2)
**Gravity irrigators**	60 (40–81)	363 (220–506)	31 (17–45)	174 (100–248)	8 (2–15)	43 (10–76)	1 (0–1)	4 (1–7)	0 (0–0)	0 (0–0)	0 (0–0)	0 (0–0)
**Semi-mechanised reseeders**	38 (29–48)	182 (136–228)	38 (32–44)	178 (151–206)	20 (22–34)	93 (62–123)	3 (1–5)	14 (6–22)	0 (0–1)	1 (0–3)	0 (0–0)	0 (0–1)
**Weeders**	31 (21–41)	142 (97–186)	43 (38–48)	192 (169–215)	21 (15–28)	95 (67–124)	4 (2–6)	19 (10–27)	0 (0–1)	2 (0–5)	0 (0–0)	0 (0–0)
